# GATA-4 and FOG-2 Expression in Pediatric Ovarian Sex Cord-Stromal Tumors Replicates Embryonal Gonadal Phenotype: Results from the TREP Project

**DOI:** 10.1371/journal.pone.0045914

**Published:** 2012-09-24

**Authors:** Calogero Virgone, Giovanni Cecchetto, Andrea Ferrari, Gianni Bisogno, Vittoria Donofrio, Renata Boldrini, Paola Collini, Patrizia Dall’Igna, Rita Alaggio

**Affiliations:** 1 Pediatric Surgery, Departments of Pediatrics and Gynaecology-Obstetrics, University Hospital, Padua, Italy; 2 Pediatric Oncology Unit, Istituto Nazionale Tumori, Milan, Italy; 3 Hematology Oncology, Departments of Pediatrics and Gynaecology-Obstetrics, University Hospital, Padua, Italy; 4 Servizio di Anatomia e Istologia Patologica, Azienda Ospedaliera di Rilievo Nazionale “Santobono Pausillipon”, Naples, Italy; 5 Pathology Unit, “Bambino Gesù” Pediatric Hospital, Rome, Italy; 6 Pathology Unit, Istituto Nazionale Tumori, Milan, Italy; 7 Pathology Unit, Department of Medical and Diagnostic Sciences and Special Therapies, University of Padua, Padua, Italy; Institut Jacques Monod, France

## Abstract

**Aim:**

GATA proteins are a family of zinc finger transcription factors regulating gene expression, differentiation and proliferation in various tissues. The expression of GATA-4 and FOG-2, one of its modulators, was studied in pediatric Sex Cord-Stromal tumors of the ovary, in order to evaluate their potential role as diagnostic markers and prognostic factors.

**Materials and Methods:**

Clinical and histological data of 15 patients, enrolled into the TREP Project since 2000 were evaluated. When available, immunostaines for FOG-2, GATA-4, α-Inhibin, Vimentin and Pancytokeratin were also analyzed.

**Results:**

In our series there were 6 Juvenile Granulosa Cell Tumors (JGCT), 6 Sertoli-Leydig Cell Tumors (SLCT), 1 Cellular Fibroma, 1 Theca Cell Tumor and 1 Stromal Sclerosing Tumor (SST). Thirteen patients obtained a complete remission (CR), 1 reached a second CR after the removal of a metachronous tumor and 1 died of disease. Inhibin was detectable in 11/15, Vimentin in 13/15, Pancytokeratin in 6/15, GATA-4 in 5/13 and FOG-2 in 11/15. FOG-2 was highly expressed in 5/6 JGCT, while GATA-4 was weakly detectable only in 1 of the cases. SLCT expressed diffusely FOG-2 (4/6) and GATA-4 (3/5). GATA-4 and FOG-2 were detected in fibroma and thecoma but not in the SST.

**Conclusions:**

Pediatric granulosa tumors appear to express a FOG-2/GATA-4 phenotype in keeping with primordial ovarian follicles. High expression of GATA-4 does not correlate with aggressive behaviour as seen in adults, but it is probably involved in cell proliferation its absence can be associated with the better outcome of JGCT. SLCTs replicate the phenotype of Sertoli cells during embryogenesis in normal testis. In this group, the lack of expression of FOG-2 in tumors in advanced stages might reveal a hypothetical role in inhibiting GATA-4 cell proliferation pathway. In fibroma/thecoma group GATA-4 and FOG-2 point out the abnormal activation of GATA pathway and might be involved in the onset of these tumors.

## Introduction

Pediatric ovarian sex cord-stromal tumors (SCST) represent a heterogeneous group of neoplasms developing from non germinative tissue. They are very rare, accounting for 5–12% of all pediatric ovarian neoplasms [1], [2].

On the basis of their histology and secretory pattern, two main groups of SCST are recognized: the granulosa-theca cell tumors and the Sertoli-Leydig cell tumors (SLCT). The granulosa-theca cell tumors represent the largest subgroup (7–8% of ovarian malignancies): although they can occur in women of any age, the typical form of the first two decades of life is the juvenile granulosa cells tumor (JGCT), frequently characterized by isosexual precocity or virilization. The Sertoli-Leydig cell tumors constitute a less frequent subgroup, representing 1–2% of all ovarian tumors. About 40% produce male hormones, causing virilization, which may be used as clinical markers. Fibrothecoma and sclerosing stromal tumor are other less frequent histotypes, generally benign [3]–[6].

The clinical behaviour varies according to the histotype: while adult granulosa cell tumors (GCT) have variable clinical presentation and outcome, juvenile granulosa cell tumors are usually confined to the ovary and do not have metastatic potential [7]. By contrast, Sertoli-Leydig cell tumors, also known as arrhenoblastomas, are huge and behave aggressively: those with retiform pattern or heterologous elements more frequently show clinical malignant course.

The histogenesis of these tumors and their relationship with their adult counterpart has been poorly investigated. In recent studies, it has been demonstrated that the expression of some factors involved in gonadal development and sexual differentiation, such as GATA-4 and FOG-2, plays a role in tumor growth and progression and might represent a prognostic factor in some sex cord-derived tumors [8], [9]. GATA proteins belong to a family of zinc finger transcription factors, which bind to a consensus GATA motif, regulating cellular gene expression, differentiation and proliferation in various tissues. GATA-1, GATA-2 and GATA-3 are involved in hematopoesis; GATA-4, GATA-5 and GATA-6 in the development of heart, endoderm, endocrine glands and gonads.

The effect of the GATA proteins is modulated through the interaction with other transcription factors, such as FOG-2 (Friend of GATA), abundantly expressed in heart, brain and testis [10], [11]. The interaction of GATA-4 with FOG-2 leads to either stimulatory or repressive modulation of transcriptional activity during the development and function of the gonads [11], [12]. The final effect comprehends the inhibition of male differentiation in the early gonad, through a subsequent action on the expression of other genes and factors, such as the Anti-Müllerian Hormone (AMH) and Steroidogenic Factor 1 (SF-1) [11], [13]–[17].

In the adult normal tissue, GATA-4 is abundantly expressed in proliferating granulosa cells of adult ovary, where it might have an antiapoptotic role, as in the fetal granulosa cells, due to its interaction with the anti-apoptotic factor Bcl-2 [16]–[19]. GATA-4 is also expressed in sex cord-derived tumors [8] and adult germ cell tumors and it has been observed that its high expression is associated with a more aggressive behaviour, therefore it represents a useful marker of poor prognosis [9]. In the group of germ cell tumors of children and young adults, GATA-4 and GATA-6 are expressed in yolk sac tumors but not in mature teratomas. This expression may have a potential role in the differentiation toward endodermal tissue and could be useful as nuclear markers in differentiating yolk sac endoderm from other components [20]–[23].

The aim of this study was to evaluate the expression of GATA-4 and FOG-2 in a series of pediatric Sex Cord-Stromal Tumors, registered into the Italian TREP project [6], in order to evaluate their potential pathogenetic and prognostic role.

The TREP project is an Italian collaborative study on very rare tumors in children [6], [25], [24] and was launched under the auspices of the Italian Society of Pediatric Surgery (SICP) and the Italian Society of Pediatric Hematology-Oncology (AIEOP). The project did not need the approval from the Ethics Committee of our Institution, because the TREP diagnostic/therapeutic guidelines were intended to be not a true protocol, but only a help in the care of these tumors: the participant institutions were not obliged to follow the TREP indications.

## Materials and Methods

The analysis includes patients ≤18 years, registered into the TREP study. Informed consent was usually taken through informed consent form and, when taken orally, the physicians in charge documented the process with a written note in the medical records of the patients. The form also included permission of the parents for data management.

Clinical and therapeutic information were obtained prospectively through special forms from the physicians in charge. The histological review was provided by the panel of pathologists from the TREP.

After the first surgical approach, the patients were grouped according to the COG/POG staging system as follows:

– Stage I. Disease limited to the ovary (or both) and completely excised; negative peritoneal washing. No clinic, surgical or histological evidence of disease extending beyond the ovary and tumoral markers’ and/or hormons’ levels in range after surgery.
– Stage II. Microscopic residuals, spillage or nodes affected by disease (<2 cm); negative peritoneal washing. Tumoral markers positive or negative.
– Stage III. Macroscopic residuals or initial biopsy only; local invasion (omentum, bowel, bladder); positive peritoneal washing; nodes affected (>2 cm). Tumoral markers positive or negative.
– Stage IV. Distant metastases. Negative or positive markers.
– Hidden disease. Stage I but tumoral markers persistently elevated after a complete surgery.

In case of incomplete excision, biopsy or metastases, a cisplatinum-based chemotherapy, plus etoposide and bleomycin, was recommended [6].

Hematoxilin/eosin sections and immunostaining (IHC) were reviewed, when available. Mitotic rate was considered high when tumor specimen displayed 10 or more mitoses/10 High Power Field (Low MI<10/10 HPF; High MI ≥10/10 HPF). Further immunostaining were performed in selected cases to confirm the diagnosis: FOG-2, GATA-4, α-inhibin, Vimentin and Pancytokeratin (MNF116) were also performed (details on IHC antibodies and techniques are showed in Table 1). FOG-2 and GATA-4 were considered positive when showing a nuclear staining. The immunostaining were scored as follows: <5% negative; 5–30% 1+; 31–60% 2+, >60% 3+.

**Table 1:** Immunohistochemistry: antibodies and techniques.

Antibody	Clone	Manufacturer	Dilution
FOG-2	Rabbit polyclonal	SANTA CRUZ	1∶50
GATA-4	Rabbit polyclonal	NOVUS BIOLOGICALS	1∶100
α-Inhibin	Clone R1	DAKO	1∶25
Vimentin	Clone V9	LEICA	1∶300
Pancytokeratin (MNF116)	Clone MNF116	DAKO	1∶200

## Results

### Clinical Features

Clinical features are summarized in Table 2.

**Table 2:** Clinical features of 15 girls affected by Sex Cord-Stromal Tumors.

CASE	AGE	STAGE	POST-SURGICALTREATMENT	HISTOLOGY	OUTCOME (Follow-Up)
**1**	31	I		JGCT with cystic predominance	CR (67)
**2**	7	I		JGCT	CR (57)
**3**	40	II		JGCT	CR (36)
**4**	73	I		JGCT	CR (72)
**5**	97	I		JGCT	CR (52)
**6**	118	I	CT (PEB)	JGCT	CR (15)
**7**	180	I		SLCT	CR (18)
**8**	144	IV	CT (PEB)	SCT-id	CR (36)
**9**	118	II	CT (PEB+IVA)	SLCT with retiform pattern and heterologous elements (primitive tumor)Prevalent sarcomatous component with focal rabdomyosarcoma-like differentiation (pelvic relapse)	DOD (33)
**10**	77	I		SCT with annular tubules	CR (15)
**11**	168	I		SCT-id with heterologous elements (1^st^ tumor)SCT-id with Leydig cell component (2^nd^ tumor)	2^nd^ CR (16)
**12**	40	I		SLCT with retiform pattern	CR (17)
**13**	175	I		Fibrothecoma	CR (24)
**14**	224	I		Fibroma (bilateral in Gorlin sdr.)	CR (84)
**15**	172	I		Sclerosing Stromal Tumor	CR (101)

Fifteen patients affected by ovarian Sex Cord-Stromal Tumors were evaluated in this study. Their age ranged from 7 to 224 months (mean 112; median 118). The staging distribution was the following: St. I 12/15, St. II 2/15, St. IV 1/15 (peritoneal spread).

In one case, the tumor (fibroma) was bilateral at diagnosis and in another case a metachronous Sertoli-Leydig cell tumor on the contralateral ovary was found two years after the initial treatment. The girl with a bilateral fibroma, who also presented a medulloblastoma at the age of five, was further investigated and a Gorlin Syndrome was diagnosed [26]. After this diagnosis she developed multiple basal cell carcinomas and odontogenic keratocysts.

Chemotherapy (cisplatin, etoposide and bleomycin) was administered in 3/15 cases: 1 St. I, 1 St. II and 1 St. IV. In one case, a different regimen (ifosfamide, vincristine, dactinomicine and doxorubicine) was delivered as second line-therapy after local and metastatic relapse (case 9).

Thirteen patients are in first complete remission, 1 obtained a second complete remission after the removal of a metachronous tumor and 1 died of disease.

**Table 3:** Pathological and immunohistochemical features.

	CASE	MORPHOLOGY	SIZE	MR/10 HPF	GATA-4	FOG-2	INH	VIM
**JGCTs**	**1**	Predominantly cystic	3 cm	<1	neg	+++	+++	++
	**2**	Solid pattern	8 cm	>10	neg	+++	++	+++
	**3**	Multiple cysts (3 cm)	10 cm	>10	neg	neg	++	++
	**4**	Multiple cysts	n.a.	21	+	+++	+++	+++
	**5**	Multiple cysts	9 cm	6	neg	+++	+++	+++
	**6**	Multiple cysts	22 cm	>10	n.a.	+++	n.a.	++
**SLCTs**	**7**	SLCT	4,5 cm	<1	+++	+++	+++	++
	**8**	SCT-id	13,7 cm	42	neg	neg	+++	+++
	**9**	SLCT; retiform pattern and heterologous elements(1^st^ tumor)Prevalent sarcomatous component withfocal RMS-like differentiation (pelvic relapse)	14 cm	>20>>20	n.a.	++	+++ (s.c.)	n.a.
	**10**	SCT with annular tubules	7 cm	1	++	++	+++	++
	**11**	SCT-id with heterologous elements (1^st^ tumor)SCT-id with Leydig cell component (2^nd^ tumor)	18 cm5,5 cm	1210	++	++	+++ (s.c.)	++
	**12**	SLCT retiform pattern	9,7 cm	4	neg	neg	+++	+++
**OTHERS**	**13**	Fibrothecoma	24 cm	<1	+++	+++	neg	+
	**14**	Fibroma (bilateral)	8 and 1 cm	<1	+++	+++	neg	++
	**15**	Sclerosing Stromal Tumor	18 cm	<1	+	neg	neg	++

### Pathological Features

Histological slides and original reports were available for all the 15 cases examined. The histological review confirmed the initial diagnosis in all cases. There were 6 juvenile granulosa cell tumors, 6 Sertoli-Leydig cell tumors, 2 fibroma/thecoma and one sclerosing stromal tumor.

Juvenile granulosa cell tumors ranged widely in diameter, from 3 to 22 cm, and appeared macroscopically as yellowish nodular lesions. Cysts were present in five cases. Microscopic examination showed well circumscribed lesions, characterized by sheets of round to slightly elongated cells, arranged in lobules separated by collagen stroma, occasionally edematous. Necrosis was generally absent, except for case 3 which also showed scattered atypical cells. Mitotic index was generally high (range:1–21 mitoses/10 High Power Field).

Sertoli-Leydig cell tumors were large tumors, from 5,5 to 22 cm, with yellow areas. Those showing Leydig cell component were darker. The group included 2 undifferentiated tumors, one of those with a rhadbomyosarcomatous component (case 11), 1 Sertoli cell tumor (SCT), 1 with annular tubules, 1 with retiform pattern, 1 a well differentiated tumor. Mitotic rate varied from 1/10 HPF to 42/10 HPF.

Fibrothecomas were white-yellowish masses, solid with focal cystic features and ranging in size from 8 to 24 cm, Microscopically they were characterized by elongated cells with clear cytoplasm, bland oval nuclei and occasional lutein cells. Mitoses were rare or absent. Bilateral fibroma showed spindle cells arranged in a prevalently collagenized stroma (case 14).

The single sclerosing stromal tumor showed cellular pseudo-lobules with blood vessels in a typical hemangiopericytomatous stroma, surrounded by fibroblasts and rounded cells, often vacuolated, with eccentric nuclei and clear cytoplasm with signet ring-like features. Mitoses were absent.

### Immunohistochemical Features

**Figure 1:**
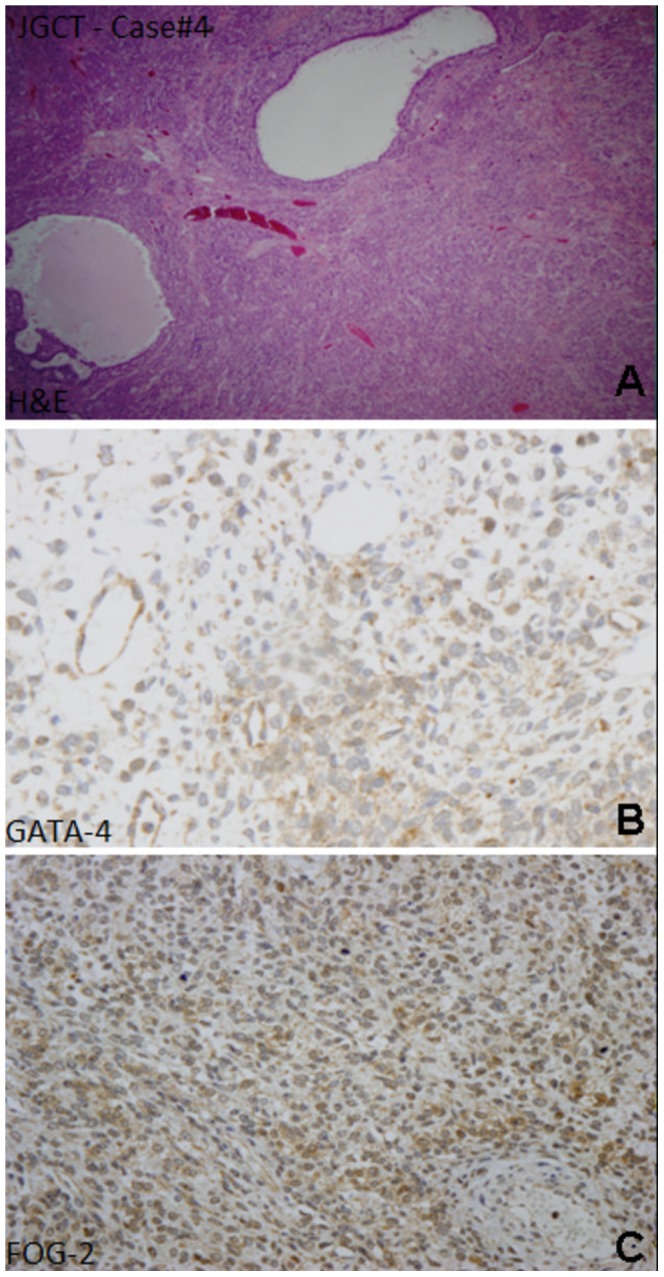
Juvenile granulosa cell tumor (A, HE stain) showing typical irregular follicles; with a profile GATA-4 positive (B) and FOG-2 positive (C).

**Figure 2:**
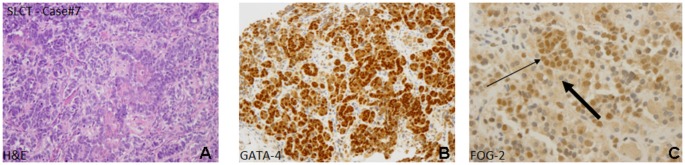
Sertoli-Leydig cell tumor with cords and Leydig cell nests (A, HE stain) with a profile GATA-4 positive (B) and FOG-2 positive: thick arrow, Leydig cells FOG-2 negative; thin arrow, Sertoli cells FOG-2 positive (C).

**Figure 3:**
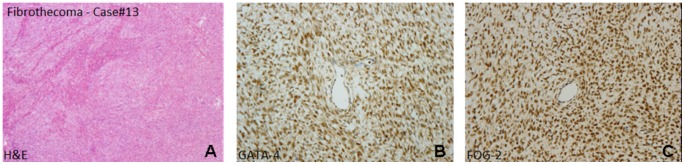
Fibrothecoma (A, HE stain) with a profile GATA-4 positive (B) and FOG-2 positive (C).

The immunohistochemical results are summarized in Table 3. Inhibin was positive in 11/15 cases, Vimentin in 13/15 and Pancytokeratin in 6/15; immunostaining for GATA-4 were positive in 5/13 and the expression of FOG-2 was detected in 11/15. In particular all juvenile granulosa cell tumors were Inhibin and Vimentin positive, whereas Pancytokeratin was focally positive in 50% (3/6). FOG-2 was highly expressed in 5/6 cases; only in a single case a weak positivity for GATA-4 was found (Figure 1). Among the Sertoli-Leydig cell tumors group (Figure 2), the expression of Inhibin, Vimentin and Pancytokeratin overlapped that of the JGCT group, with a strong positivity for Inhibin (5/6) and Vimentin (5/6). FOG-2 was diffusely expressed also in these tumors (4/6) and a positivity for GATA-4 was found in 3/5 cases. In the other less frequent histotypes (2 fibroma/thecoma and 1 SST), Inhibin was negative in 3/3, FOG-2 was detected in both fibroma/thecoma and resulted negative in the sclerosing stromal tumor. GATA-4 was positive in 2/2 fibroma/thecoma (Figure 3).

## Discussion

Because of the rarity of Sex Cord-Stromal Tumors, only a few studies investigated their biological characteristics in children. The vast majority of data comes from previous experiences in adults, in whom, however, these tumors are characterized by different clinical behaviour and histology.

In children, the completeness of resection, favoured by an early diagnosis, and histology are important prognostic factors. In our series, all patients who underwent an initial complete excision were cured, independently from tumor size [6]. Our previous study confirmed a favourable clinical course in the majority of SCST, and a more aggressive behaviour of Sertoli-Leydig cell tumors.

The diagnosis of SCST relies essentially upon morphology, while immunohistochemistry plays a minor role. As previously reported, Inhibin is expressed in the vast majority of cases, except in fibroma-thecoma, where it may be negative [27], [28]; GATA-4 has been recently found to be expressed in adult ovarian GCT and in testis SLCT [8], [29] and to be associated with an aggressive behaviour in adult Granulosa Cell Tumors [9].

In the present series, the immunohistochemical pattern of GATA-4 and FOG-2 expression differed among the various histotypes. Intriguingly, juvenile granulosa cell tumors, the more indolent subtype, were mostly negative for GATA-4, but showed a strong expression of FOG-2. This is in contrast with adult GCTs, which have been reported to co-express FOG-2 and GATA-4 in most cases [9]. This apparent contrast may be explained by a different histogenesis of these tumors, reproducing the JGCT the phenotype of primordial follicle during fetal life, and the adult granulosa cell tumors the normal primary follicle [8].

**Figure 4:**
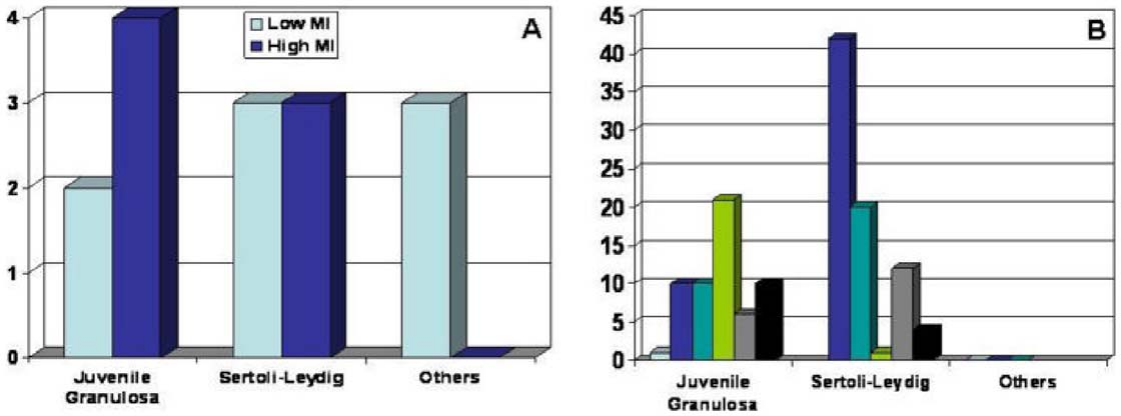
Bar charts showing Mitotic Rate for each Sex Cord-Stromal Tumor subgroup (A, High MI tumors and Low MI tumors for each histological subtype; B, MI for each case grouped by histology).

It might be speculated that the overall good prognosis of JGCT can be related to a different genetic pathway with repression of GATA-4, as confirmed by the high levels of FOG-2. To further support this hypothesis, we observed that the only tumor presenting a weak positivity for GATA-4 (case 4) showed a mitotic activity superior to 21 mitoses/10 HPF, although none of JGCTs of our series could be considered clinically malignant and considering that mitotic rate is not prognostically relevant in low stage JGCT [2]. The good outcome of low stage JGCT, although showing a high mitotic rate (Figure 4), may be probably explained not only by the inhibiting activity of FOG-2, abundantly expressed in most JGCTs of this series, but also by an early diagnosis and prompt excision.

The phenotypic overlap between embryonic gonad and tumors was observed also in Sertoli-Leydig cell tumors: GATA-4 and FOG-2 pattern of expression replicates the phenotype of Sertoli cells during embryogenesis in the normal testis in which FOG-2 is weakly expressed and GATA-4 more frequently positive [13], [29]. In the human testis GATA-4 is expressed from early gonadal development to adulthood: in Sertoli cells it is evident through fetal and postnatal development, whilst in Leydig cells it is expressed during fetal period and after puberty, coinciding with the phases of active androgen synthesis in the testis. FOG-2 expression has been demonstrated to decrease with the progression of gonadal development [13], [29].

High GATA-4 expression in tumoral cells was reported in a pediatric series of 1 testicular Sertoli cell tumor and 5 testicular Leydig cell tumors: this expression was higher in neoplastic cells than in normal adjacent Sertoli cells and Leydig cells of the same patients [29].

This increased expression of GATA-4 in Sertoli-Leydig cell tumors in comparison to juvenile granulosa cell tumors did not seem to be related to a more aggressive behaviour as expected: there was no apparent correlation between high mitotic rate and GATA-4 expression, whereas detection of a high FOG-2 expression could be linked to a lower tumor stage at diagnosis. These data suggest that FOG-2 might maintain its role as GATA-4 downregulator even in tumoral cells. This has already been suggested by Mannisto and colleagues who observed that in a series of yolk sac tumors the aggressive clinical behaviour was related to the higher activity of GATA-4 in the absence of FOG-2 [22].

In the thecoma/fibroma group the expression of both FOG-2 and GATA-4 might be useful in differential diagnosis from other spindle cell tumors: 2/3 (cases 13 and 14) showed high expression of both GATA-4 and FOG-2, and the sclerosing stromal tumor (case 15) showed an intermediate positivity for GATA-4. Although the scarcity of cases in this group prevents to draw assured conclusions, some hints can be underlined. GATA-4 and FOG-2 are expressed in normal embryonal stroma: in adult tissues of mesenchymal origin, GATA-4 is less expressed and FOG-2 is lacking [8], [16]. On the contrary, their concomitant strong expression might indicate an abnormal activation of the GATA pathway of cellular proliferation and immortalization: GATA-4 may be co-responsible of the progression of these neoplasms, though they are indolent and clinically benign. On the other hand, the high expression of FOG-2 might indicate once again an inhibiting action on GATA-4-mediated proliferation and antiapoptotic activity, thus partially explaining the low mitotic rate of this subgroup.

In conclusion, our study on Sex Cord Stromal Tumors in childhood showed a possible switch to embryonal gonadal phenotype: respectively, juvenile granulosa cell tumors appeared to express a FOG-2/GATA-4 pattern in keeping with primordial ovarian follicles and Sertoli-Leydig cell tumors with embryonal testis.

The overall good prognosis of juvenile granulosa cell tumors is consistent with their low expression of GATA-4: this factor is probably involved in cell proliferation and its absence may be linked to the better outcome of JGCT. Sertoli-Leydig cell tumors, which represent the more aggressive histotype, showed a higher expression of GATA-4, conversely to what detected in JGCTs. Nevertheless, this data did not seem to have a prognostic value by itself. FOG-2 might have a more relevant prognostic value: its absent expression in advanced stage tumors, and also its higher expression in low stage and low grade tumors, may reveal a hypothetical role in inhibiting GATA-4 cell proliferation pathway.
